# Platinum Nanoparticles in Biomedicine: Preparation, Anti-Cancer Activity, and Drug Delivery Vehicles

**DOI:** 10.3389/fphar.2022.797804

**Published:** 2022-02-23

**Authors:** Atena Abed, Maryam Derakhshan, Merat Karimi, Matin Shirazinia, Maryam Mahjoubin-Tehran, Mina Homayonfal, Michael R Hamblin, Seyed Abbas Mirzaei, Hamidreza Soleimanpour, Sadegh Dehghani, Farnaz Farzaneh Dehkordi, Hamed Mirzaei

**Affiliations:** ^1^ Department of Medical Biotechnology, School of Advanced Technologies, Shahrekord University of Medical Sciences, Shahrekord, Iran; ^2^ Cellular and Molecular Research Center, Basic Health Sciences Institute, Shahrekord University of Medical Sciences, Shahrekord, Iran; ^3^ Department of Pathology, Isfahan University of Medical Sciences, Kashan, Iran; ^4^ Institute of Nanoscience and Nanotechnology, University of Kashan, Kashan, Iran; ^5^ Faculty of Medicine, Mashhad University of Medical Sciences, Mashhad, Iran; ^6^ Department of Medical Biotechnology, Faculty of Medicine, Mashhad University of Medical Sciences, Mashhad, Iran; ^7^ Research Center for Biochemistry and Nutrition in Metabolic Diseases, Institute for Basic Sciences, Kashan University of Medical Sciences, Kashan, Iran; ^8^ Laser Research Centre, Faculty of Health Science, University of Johannesburg, 2028 Doornfontein, Johannesburg, South Africa; ^9^ Department of Medical Biotechnology, National Institute of Genetic Engineering and Biotechnology, Tehran, Iran; ^10^ Pharmaceutical Research Center, Pharmaceutical Technology Institute, Mashhad University of Medical Sciences, Mashhad, Iran; ^11^ Department of Biology, Ardabil Branch, Islamic Azad University, Ardabil, Iran; ^12^ Student Research Committee, Kashan University of Medical Sciences, Kashan, Iran

**Keywords:** platinum nanoparticles, cancer therapy, delivery systems, nanoparticle, cancer

## Abstract

Cancer is the main cause of morbidity and mortality worldwide, excluding infectious disease. Because of their lack of specificity in chemotherapy agents are used for cancer treatment, these agents have severe systemic side effects, and gradually lose their therapeutic effects because most cancers become multidrug resistant. Platinum nanoparticles (PtNPs) are relatively new agents that are being tested in cancer therapy. This review covers the various methods for the preparation and physicochemical characterization of PtNPs. PtNPs have been shown to possess some intrinsic anticancer activity, probably due to their antioxidant action, which slows tumor growth. Targeting ligands can be attached to functionalized metal PtNPs to improve their tumor targeting ability. PtNPs-based therapeutic systems can enable the controlled release of drugs, to improve the efficiency and reduce the side effects of cancer therapy. Pt-based materials play a key role in clinical research. Thus, the diagnostic and medical industries are exploring the possibility of using PtNPs as a next-generation anticancer therapeutic agent. Although, biologically prepared nanomaterials exhibit high efficacy with low concentrations, several factors still need to be considered for clinical use of PtNPs such as the source of raw materials, stability, solubility, the method of production, biodistribution, accumulation, controlled release, cell-specific targeting, and toxicological issues to human beings. The development of PtNPs as an anticancer agent is one of the most valuable approaches for cancer treatment. The future of PtNPs in biomedical applications holds great promise, especially in the area of disease diagnosis, early detection, cellular and deep tissue imaging, drug/gene delivery, as well as multifunctional therapeutics.

## Introduction

Cancer is the main cause of human mortality excluding infectious disease (Siegel et al., 2021; Sung et al., 2021). Conventional chemotherapeutic agents and anti-cancer drugs such as anti-metabolites and alkylating agents have many toxic effects on patients. Therefore, researchers are interested in utilizing targeted therapy to decrease their side effects (Shafabakhsh et al., 2019; Gao and Dong, 2020). The use of nanomaterials is a new approach to control the release of anti-cancer drugs and increase their *in vivo* efficacy. Nanoparticles (NPs) can be used as anti-cancer drug carriers. Metal-based NPs can have intrinsic anti-tumor effects, including an antioxidant action (Yu et al., 2022). Furthermore, the anti-cancer activity of NPs can be improved by the use of external energy sources, for example hyperthermia can be produced by applying magnetic fields or infrared radiation (Tseng et al., 2014; Bloch et al., 2021). When NPs are stimulated with external energy, they can produce reactive oxygen species which can destroy the cancer cells and also interact with the tumor environment, including stroma and blood vessels, and inhibit tumor development (Kankala et al., 2020; Bloch et al., 2021; Saitoh et al., 2021). Metallic NPs are increasingly being used in biomedical research. In the case of cancer, they can be designed for therapeutic as well as diagnostic applications (Fu et al., 2020; Kankala et al., 2020; Mukherjee et al., 2020; Bloch et al., 2021; Saitoh et al., 2021; Yu et al., 2021). Metallic and metal oxide NPs can be produced and modified with a variety of chemical functional groups as needed. They can be conjugated with biological molecules (antibodies, nucleic acids or peptides), targeting ligands, and anticancer drugs using appropriate functionalization strategies (Fu et al., 2020; Oliveira et al., 2020; Patel et al., 2020; Zeng et al., 2020). Herein, we summarize the preparation, characterization, and anti-cancer effects of platinum nanoparticles (PtNPs). Moreover, we highlight the use of PtNPs as delivery systems in cancer therapy.

## PtNPs Preparation Methods

**FIGURE 1:**
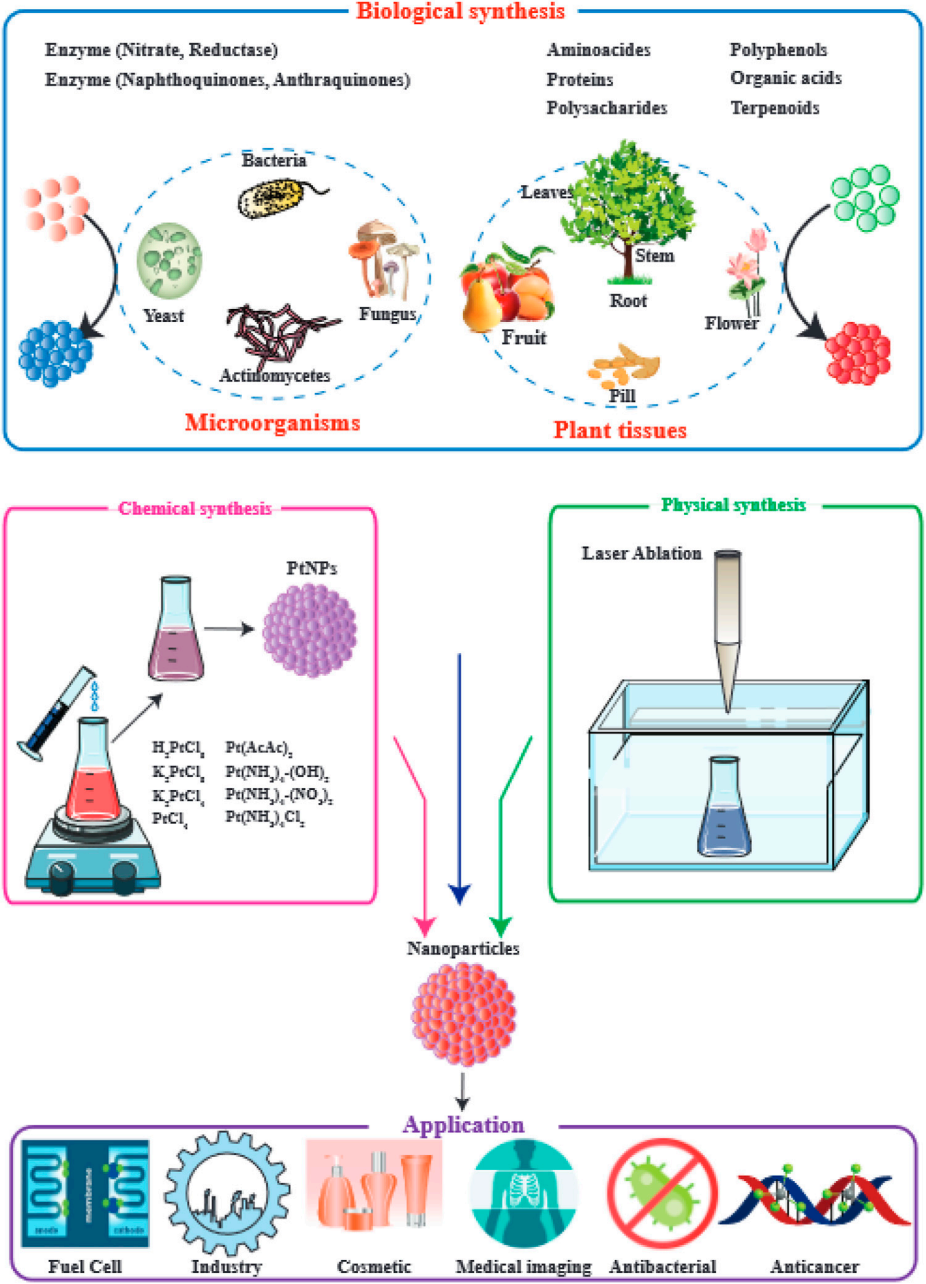
Applications of PtNPs and their methods of preparation. This figure adapted from Jeyaraj et al. (2019).

The overall synthetic approaches for PtNPs include physical methods, chemical methods, and biological methods as shown in Figure 1.

### Physical Methods

In physical methods a top-down approach is used. In the top-down approach, various physical techniques can generate NPs through mechanical degradation of the structure of bulk metallic materials. This approach requires energy and is an overall economic process but also takes a long time. However, it may be the best way to regulate the size range of NPs and control their morphologies. The top-down method starts with the bulk material, which is progressively degraded, leading to the generation of fine NPs. For mass manufacture of NPs, a variety of physical techniques are used, including photolithography, milling techniques, anodization, and electron beam lithography (Fichtner et al., 2020; Garlyyev et al., 2020; Kuriganova et al., 2020).

Top down production of PtNPs involves the administration of thermal energy, electrical energy, high energy radiation, or mechanical pressure, leading to the abrasion of bulk metal, or melting, evaporation and condensation in order to synthesize NPs. Thermal techniques to produce PtNPs involve heating in a tube furnace at high air pressure, leading to evaporation followed by condensation. Physical methods have the following advantages: rapidity and purity, lack of toxic chemical reagents, uniform size and shape; however, they have the disadvantages of low productivity, high cost, radiation exposure, high pressure, high temperature, energy consumption, lower thermal stability, significant waste products, high dilution, inadequate control of shape and size. This technique is ineffective for producing NPs with controllable and uniform shapes and sizes, and it significantly alters the physicochemical architecture and the surface of the NPs. There are different types of physical methods which have been used, including; laser ablation, arc discharge, flame spray pyrolysis, ball milling, melt mixing, vapor deposition, and sputter deposition (Dhand et al., 2015).

#### Laser Ablation

Laser ablation is a simple but expensive process that involves irradiating a solid (or occasionally a liquid) surface with a laser beam to gradually remove material and can be used as an alternative to electrical heating. Laser ablation involves high costs to produce sufficient energy, but its overall energy efficiency is good. Materials exposed to a laser flux absorb the incident laser energy, turning the material to plasma. Laser ablation of solid metal has problems with aggregation and inadequate degradation, therefore in previous studies many researchers have shown generation of PtNPs by PLAL (pulsed laser ablation in liquid) (Waag et al., 2021; Fedorova et al., 2020; N Madlum et al., 2021). The milling process involves gradually decreasing the particle size and mixing them into new phases (Jan et al., 2021; Mathew et al., 2021).

#### Solvothermal Processes

In solvothermal processes, reactions are carried out at a lower temperature because the reactants become more soluble. It is a low-temperature method that uses polar solvents at high pressure and at temperatures greater than their normal boiling points (Jameel et al., 2021; Mathiesen et al., 2021).

#### Inert Gas Condensation

IGC methods are employed to evaporate metals in vacuum chambers containing an inert gas at a usual pressure of 100 Pa. IGC is a very efficient process for producing high-quality silver NPs and PtNPs (Maicu et al., 2014). Collisions between gas atoms and metal atoms occur in IGC, and the vaporized metal atoms lose kinetic energy and undergo condensation to produce tiny crystals that accumulate in liquid nitrogen. The same method is also employed for the preparation of gold NPs.

### Chemical Methods

The production of metal NPs in chemical solutions *via* diverse chemical reactions is defined as chemical preparation. The bottom-up method is used in the chemical production of NPs. The bottom-up approach allows individual atoms and molecules to self assemble into various sizes of NPs (Fahmy et al., 2020a; Dang‐Bao et al., 2021). The following are examples of the bottom-up approach (Sung et al., 2021): self-assembly of monomer/polymer molecules (Siegel et al., 2021); chemical or electrochemical nanostructured precipitation (Gao and Dong, 2020); sol–gel formation (Shafabakhsh et al., 2019); laser pyrolysis (Yu et al., 2022); chemical vapor deposition (CVD) (Bloch et al., 2021); plasma or flame spraying synthesis (Tseng et al., 2014); bio-assisted synthesis (Nichols et al., 2006).

**FIGURE 2:**
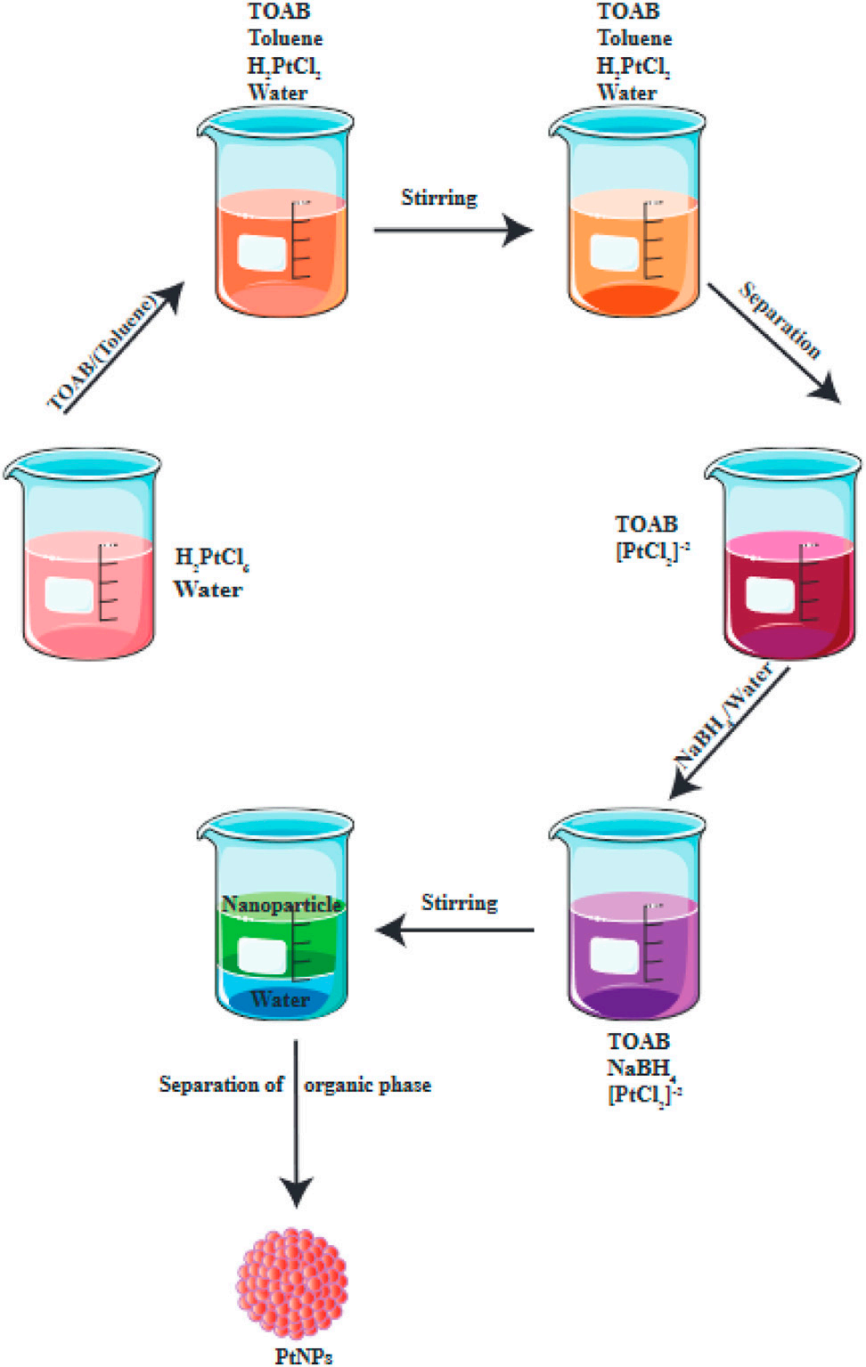
Chemical reduction is used for the production of PtNPs. This figure adapted from Jeyaraj et al. (2019).

One chemical process is known as nucleation, and it primarily requires the use of water-soluble metal cations as a precursor that can be reduced to form metal atoms (Quinson and Jensen, 2020). The process of capping stops the reducing agent, and blocks the growth of the reduced metal atom cluster at the NP scale. Chemical reduction is particularly employed to generate colloidal metal NPs, where chemical agents reduce the metallic ions, leading to formation of metallic NPs (Figure 2). Particles may become thermally stable once their shape has reached a specific size (Quinson and Jensen, 2020).

For the production of PtNPs, a variety of conditions must be optimized, including appropriate temperature, reducing/capping agent, as well as size and shape (Brown et al., 2021). Many studies have optimized the reagents and conditions for the production of PtNPs (Shukla et al., 2007; Du et al., 2008; Yamada et al., 2015). A reducing agent, a capping/stabilizing agent and the metal precursor comprise the three essential components for PtNP preparation. Moreover, aggregation should also be avoided in the approach. Chemical techniques have the following advantages: low cost, good surface chemistry flexibility, simple functionalization, high yields, good control of size, thermal stability, decreased dissipation. However the disadvantages are: low purity, use of toxic chemical reagents and organic solvents that may be harmful to humans and the environment (Quinson and Jensen, 2020; Jan et al., 2021; Mathiesen et al., 2021).

#### Reduction in Nonpolar Solvents

Chemical reduction of metal ions inside reversed micelles in a nonpolar solvent is the most well-known technique to generate metal NPs (MNPs) (Stepanov et al., 2014). A metal salt dissolved in water is first incorporated in reverse micelles, and is then chemically reduced to produce MNPs. Because size of the particles is important, the volume of the reverse micelles and also the ratio of water to solvent should be carefully controlled.

#### Fusion Approach

In the early 1920s, Adam and his colleagues produced bulk type PtO_2_ by a fusion approach at 450°C (Adams, 1974). The material was then modified into PtNPs using a variety of techniques. However these techniques often use toxic chemicals.

#### Wet Chemical Reduction

**FIGURE 3:**
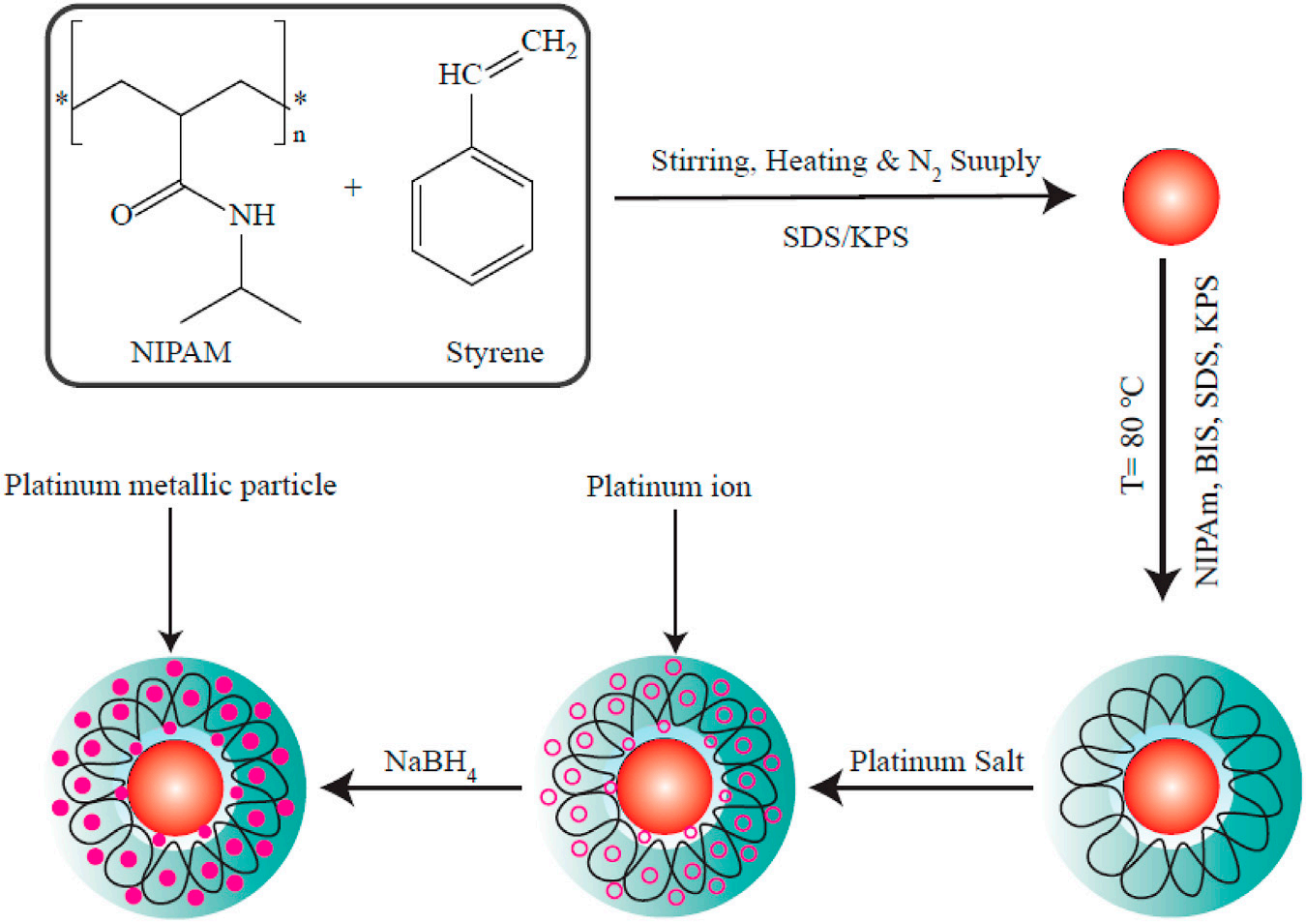
Chemical synthesis of doped platinum NPs.

Wet chemical reduction is frequently employed to better regulate the size of particles. Numerous chemical reducing agents can be used, including methoxy polyethylene glycol, sodium borohydride, trisodium citrate dihydrate, potassium bitartrate, elemental hydrogen, and ascorbate. The size and shape of the produced NPs depends on the temperature (Leong et al., 2014), the reducing agent (Tao et al., 2008; Chen et al., 2009; Lim et al., 2010; Miyabayashi et al., 2011), and the concentration of the precursor platinum compound (Figure 3).

#### Other Chemical Methods

A wide range of other chemical synthesis methods have been used, including sol–gel process, pyrolysis, chemical vapor deposition (CVD), microemulsion, hydrothermal, polyol synthesis, electrochemical process, photochemical reduction, hydrolysis, thermal decomposition, confined reaction, sono-decomposition, chemical vapor deposition, and plasma-enhanced chemical vapor deposition (Bönnemann and Richards, 2001), (Liu et al., 2002; Zeng et al., 2006; Kim et al., 2013) (Abdolhosseinzadeh et al., 2018), (Meltzer et al., 2001) (Reetz and Koch, 1999), (Du et al., 2006), (Fujimoto et al., 2001), (Okitsu et al., 2000), (Saminathan et al., 2009), (Shafiei et al., 2010).

### Biological Methods

#### Green Synthesis of PtNPs Using Plant Extracts

Plant derivatives include a variety of primary and secondary compounds that can act as natural reducing agents and capping agents (Bhattacharya and Gupta, 2005; Mandal et al., 2006; Ahmad et al., 2010; Mukunthan and Balaji, 2012; Kharissova et al., 2013; Puja and Kumar, 2019; Castillo-Henríquez et al., 2020). Plant derivatives have been used in the green synthesis of various MNPs, according to several reports (Shankar et al., 2004; Kasthuri et al., 2009; Azzazy et al., 2012). Biosynthesis of MNPs mediated by plants is a simple and rapid process that involves a combination of a plant derivatives and a solution of metal ions at optimal pH and temperature values. A color change in the reaction medium indicates the formation of the NPs (Marslin et al., 2018). Temperature, pH and reaction should all be adjusted to regulate the average shape, size and surface charge of the MNPs (Sathishkumar et al., 2010; Castro et al., 2011; Sankar et al., 2014; Wang et al., 2020). Despite the fact that the reaction mechanism of the biosynthesis of MNPs using plant derivatives needs to be comprehensively explored, a bottom-up mechanism has been postulated (Durán et al., 2011; Keat et al., 2015). There are four essential steps in this hypothesized mechanism: (Sung et al., 2021) the initial activation step (bio-reduction), where metal ions undergo reduction to produce a zero-oxidation state (Malik et al., 2014); (Siegel et al., 2021) the second step involves development and aggregation of these tiny particles into NPs with higher thermal stability (Gao and Dong, 2020); the third stage (termination), in which the MNPs are stabilized and capped to produce NPs with a controlled range of shape and average size (Malik et al., 2014; Fahmy et al., 2020b); (Shafabakhsh et al., 2019) the final stage which involves washing and purification of the MNPs often by centrifugation (Fahmy et al., 2020b).

**FIGURE 4:**
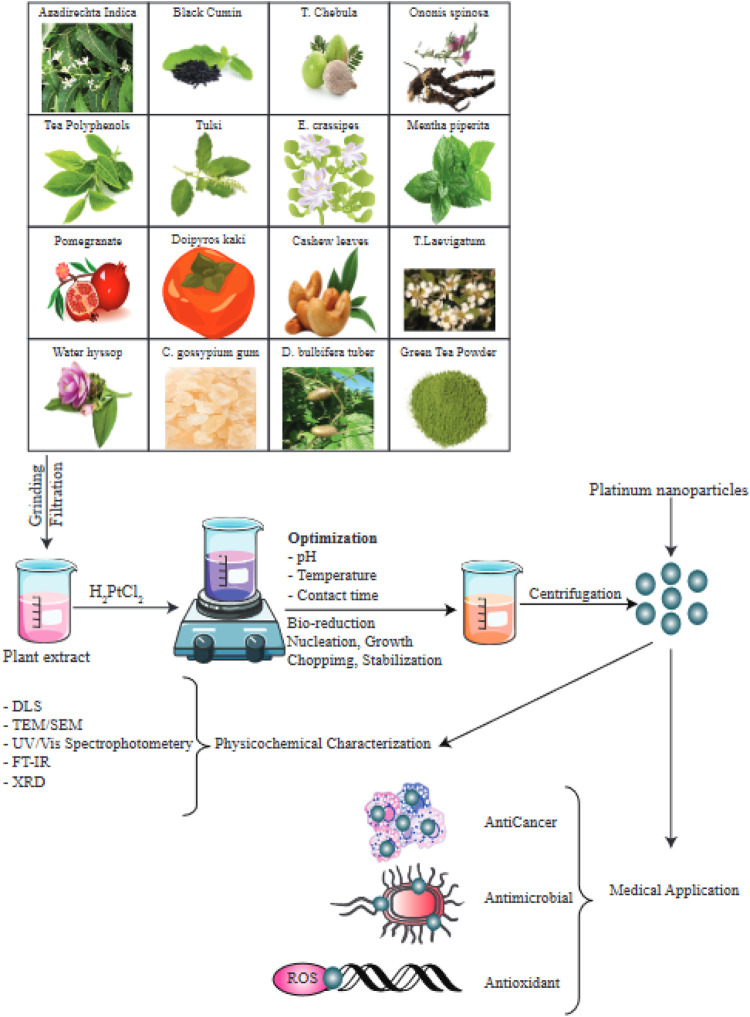
Numerous species of plants have been used in bioreduction procedures to produce PtNPs. The factors optimized as well as the methods used to characterize the PtNPs are depicted in this diagram. This figure adapted from Jeyaraj et al. (2019).

By regulating the temperature, reaction time, and pH, researchers can optimize the size, morphology. and crystallinity of the MNPs (Feldheim and Foss, 2002; Shahverdi et al., 2011). MNPs in general and PtNPs in particular, are characterized using UV/Vis spectrophotometry, scanning electron microscopy, TEM (transmission electron microscopy), FTIR (Fourier transform infrared spectroscopy), and powder XRD (X-ray diffraction) (Kumar et al., 2004; Nath and Banerjee, 2013; Malik et al., 2014; Mashwani et al., 2016; Khan et al., 2017; Nadimpalli et al., 2018; Aygun et al., 2020). Various plant species have been used in the green production of PtNPs as shown in Figure 4.

#### Synthesis of PtNPs Using Microorganisms

Both single-celled and multicellular organisms are capable of producing inorganic materials either extracellularly or intracellularly (Nath and Banerjee, 2013). Overall, MNPs could be generated by bacteria using a reduction process governed by intracellular signaling pathways, in which bacterial enzymes converting metal ions into MNPs. The simplicity of handling is one of the main benefits of bacteria-based NP production. Genetic engineering methods can also be used to modify the bacterial cells for specific objectives, such as reducing the toxicity and allowing long-term NP production in cultures. This approach also has certain drawbacks, such as being a time-consuming procedure, high costs, needs downstream processing, and a lack of control over the size and shape of the product. Metal NPs such as silver, for example, have been produced as external bacterial products or as intracellular extracts from bacteria (Kalimuthu et al., 2008; Kalishwaralal et al., 2008). The downstream procedure required for the separation of AgNPs from cell-derived extracts takes a long time. In a study by Riddin et al. the biosynthesis of geometric PtNPs employing soluble, cell-free protein derivatives from a bacterial strain with sulfate-reducing capability was compared to the use of whole cells of the same culture, leading to production of amorphous Pt^(0)^ NPs (Riddin et al., 2010). PtNPs have been synthesized by bacteria with sulfate-reducing capability such as Desulfovibrio desulfuricans (Martins et al., 2017) or *Acinetobacter* calcoaceticus (Gaidhani et al., 2014). These sulfate-reducing bacteria have the potential to successfully reduce platinum (IV) ions into platinum^(0)^ NPs produced over 24 h, at temperatures below 30°C and pH 7.0 showed the highest production yield. The NPs showed a cuboidal morphology with a size of 2–3.5 nm. These PtNPs were precipitated from Shewanella algae (a bacterial strain with metal-ion reducing potential), and the remainder of the PtNPs were deposited by adding the reducing ion PtCl_6_^2−^ to produce elemental platinum at a neutral pH and room temperature. NPs with a size of 5 nm were shown to be located within the periplasmic space of S. algae cells. This biological process could have two main mechanisms, uptake and deposition or else incorporation (Konishi et al., 2007).

Various fungal species have been employed in the biosynthesis of MNPs. In comparison to plants or prokaryocytes, the use of fungi in the production of MNPs has some advantages, since NPs with a more uniform shape and size, and well-defined dimensions are generated. In addition, fungi grow in a relatively simple medium, allowing easy downstream processing, and scale-up production. Fungi create an easy-to-handle biomass and can secrete significant amounts of protein (Riddin et al., 2006; Govender et al., 2009; Syed and Ahmad, 2012) so that reducing enzymes can generate the NPs (Mohanpuria et al., 2008). Subsequently, NPs with high stability are generated and their aggregation may be prevented (Mukherjee et al., 2008; Narayanan and Sakthivel, 2010). In conclusion, researchers have investigated the use of fungi as an appropriate source of nanomaterials. The majority of fungi generate MNPs *via* either extracellular or intracellular pathways. NPs produced *via* an extracellular process, are non-toxic and allow adequate commercial availability.

## PtNPs as Therapeutic Agents in Cancer Therapy

PtNPs have several uses, including cancer diagnosis and therapy, as well as overcoming multi-drug resistance in bacteria (Jabir et al., 2012). The biomedical applications of nanotechnology may not only overcome bacterial antibiotic resistance, but can also help in the management of tumors (Taylor and Webster, 2011). By including hydrophilic polymers which provide a suitable surface for opsonization, MNPs can be used in cancer treatment (Jabir et al., 2012).

**FIGURE 5:**
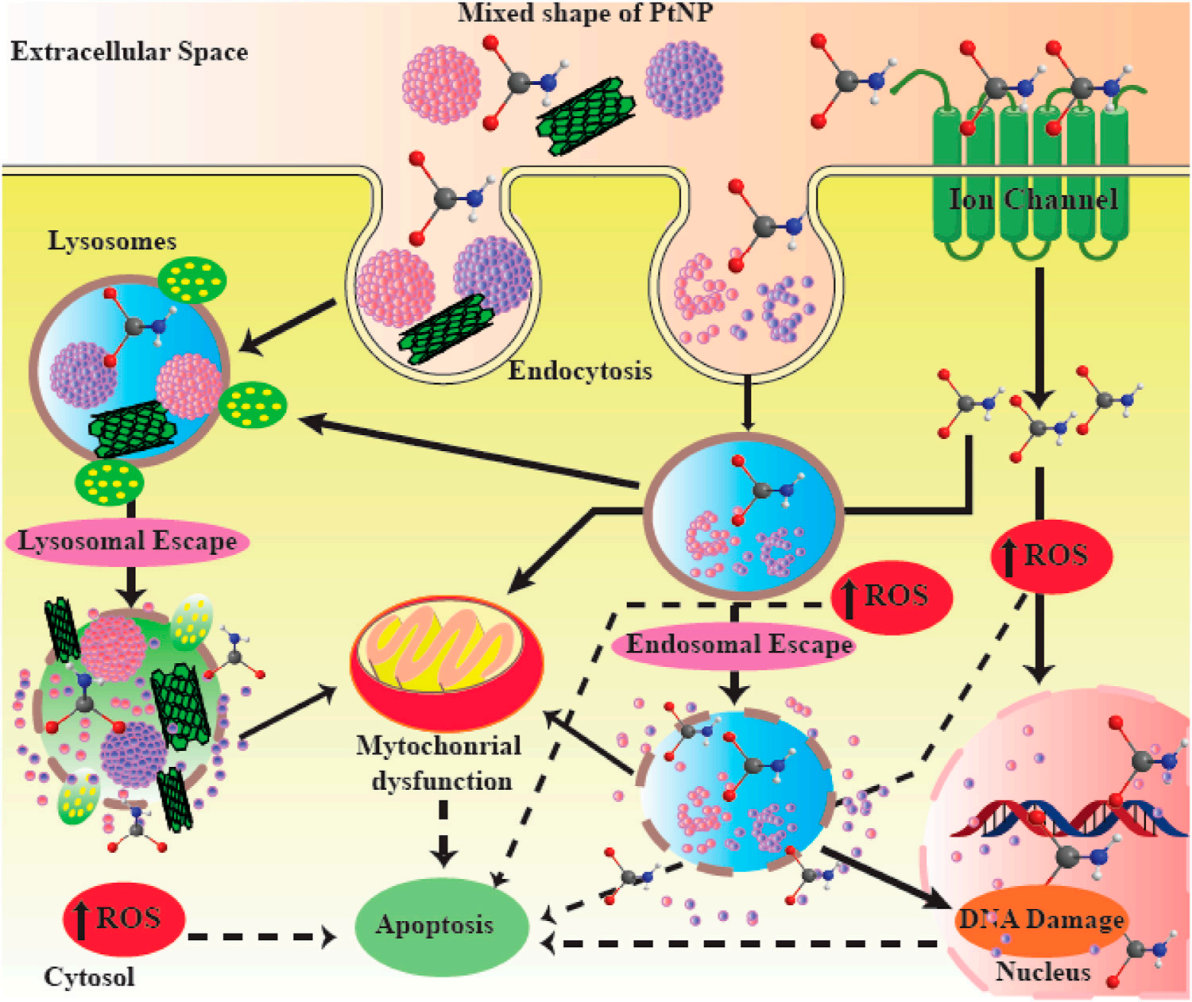
Possible mechanisms of action of PtNPs. PtNPs are able to induce apoptosis and DNA damage in cancer cells. This figure adapted from Jeyaraj et al. (2019).

Platinum itself has a strong anticancer effect (Reedijk, 1987). PtNPs behave differently compared to platinum-containing compounds, but they possess similarly efficient anticancer activity. After entering the cell through passive diffusion or by endocytosis, PtNPs exert cytotoxicity depending on their size, concentration and incubation time, This is mainly caused by the introduction of strand breaks in the chromosomal DNA (Figure 5) (Porcel et al., 2010; Johnstone et al., 2016; Kutwin et al., 2017). DNA damage leads to the inhibition of replication, and the induction of cell cycle arrest and apoptosis. Another possible mechanism of action of PtNPs involves inhibition of cellular metabolic activity, generation of hydroxyl radicals, and release of active free Pt^2+^ ions, a strategy which is currently used in radiotherapy. At certain concentrations, PtNPs can also act as antioxidants (Asharani et al., 2010; Gehrke et al., 2011; Prasek et al., 2013). In addition, some reports have proposed the potential use of PtNPs as coatings for sensors to detect glucose or other biomolecules (Taurino et al., 2015). Moreover, PtNPs can augment the host anti-tumor immune response through enhanced antigen presentation and T cells activation (Yu et al., 2022).

PtNPs can be also used for diagnosis and imaging. For instance they exhibit good photostability after multiphoton stimulation inside cells, therefore they can be used for imaging and microscopy (Botchway et al., 2008). In multiphoton imaging, the depth is improved by utilizing light with a longer wavelength and a lower energy level in the visible or near infrared spectral regions (Botchway et al., 2008). Platinum NPs are a good alternative because of their reduced toxicity and favorable imaging properties (Bendale et al., 2017).

A unique silver (Ag)-platinum (Pt) NP dendritic assembly was prepared by López Ruiz et al. (2020a). TEM analysis was used to characterize the NPs, and EDX was used to confirm their composition. Gram-negative *Pseudomonas aeruginosa*, Gram-negative multi-drug resistant *Escherichia coli* and Gram-positive *Staphylococcus aureus* were all employed to test the antibacterial activity. To assess anticancer activity, researchers used two different cancer cell lines, glioblastoma and melanoma. Finally, cytotoxicity was assessed using normal fibroblasts. The TEM examination showed the AgPtNPs had a dendrimeric NP shape with a mean size of 42 ± 11 nm. The presence of both Ag and Pt metals was confirmed by EDX analysis of the elemental composition. Medically relevant pathogenic bacteria *P. aeruginosa*, *E. coli,* and *S. aureus* were substantially inhibited by the AgPt NPs, which were more effective than silver NPs. The MTS test demonstrated a dose-dependent (10–250 g/ml concentration range) and selective anti-tumor activity of AgPt NPs against melanoma and glioblastoma cell lines. At doses ranging from 10 to 50 g/ml, cytotoxicity tests with normal fibroblasts did not show any harmful effects of NPs on normal cells. These AgPtNPs warrant further investigation as potential anticancer and antibacterial agents (López Ruiz et al., 2020a).

Baskaran et al. reported the use of *Streptomyces spp* to synthesize PtNPs in an environmentally friendly manner (Baskaran et al., 2017). The generated PtNPs were found to have a face centered cubic system. The involvement of protein amino acids, which act as a crucial reductant for the production of PtNPs, was shown by the FT infrared spectrum. Topographical images of field emission scanning electron microscopy and atomic force microscopy showed the spherical shape of PtNPs with an average size of 20–50 nm. The higher purity of the PtNPs was confirmed by X-ray fluorescence spectra. PtNP size was verified using transmission electron microscopy, and the particles were found to have a uniform size range. PtNPs also exhibited a typical surface plasmon resonance peak at 262 nm. Dynamic light scattering showed that 97.2% of particles were less than 100 nm, with an average particle diameter of 45 nm. Using the 3-(4, 5-dimethyl-2-thiazolyl)-2, 5-diphenyl-tetrazolium (MTT) assay *in vitro*, PtNPs exhibited an IC50 of about 31.2 μg/ml against MCF-7 breast cancer cells (Baskaran et al., 2017).

Alshatwi et al. used tea polyphenol (TPP) as a surface modification and reducing agent to generate PtNPs (Alshatwi et al., 2015). TEM and XRD were utilized to evaluate the shape and crystalline nature of the TPP-functionalized PtNPs (TPP@Pt). The TPP@Pt had a crystalline nature with a face-centered cubic structure, according to the XRD results. TTP@Pt were flower-shaped, according to TEM imaging, with a well-dispersed 30–60 nm-sized structure. TPP@Pt was then used to treat cervical cancer cells (SiHa) at various doses. Cell cycle progression, nuclear shape, and cell viability were measured. TPP@Pt reduced the growth of SiHa cells, according to a cell culture experiment. Nuclear staining with propidium iodide revealed that TPP@Pt caused chromatin condensation and nuclear fragmentation. Treatment with TPP@Pt increased the proportion of cells in the G2/M stage, indicating cell cycle arrest, as well as an increased number of cells the sub-G0 cell death stage. These data suggested that TPP@Pt might be used to treat cervical cancer (Alshatwi et al., 2015).

Satraplatin, is a platinum-based medicinal drug, previously shown to cause apoptosis by arresting the cell cycle at the G2/M phase (Kalimutho et al., 2011). Shingu et al. also created a platinum compound with two Pt atoms connected together by a spermine linker that led to G2/M cell cycle arrest followed by apoptosis (Shingu et al., 2010). According to previous studies, cell cycle arrest at G2/M phase following DNA injury can result in different outcomes, such as cellular apoptosis, longer permanent arrest, or recovery after DNA damage by repair or adaptation to the damage, which restarts cell cycle progression, while the DNA damage is still present (Passalaris et al., 1999). Cells that undergo G2 arrest may continue and progress their cell cycle or die by apoptosis (Sorenson et al., 1990).

Gurunathan et al., explored the effects of PtNPs on the exosomal and cellular functions in A549 (human lung epithelial adenocarcinoma cells) (Gurunathan et al., 2021a). In A549 cells, PtNPs altered several physiological processes leading to the increased secretion of exosomes. Moreover, in A549 cells cultured with PtNPs, the overall amount of protein biosynthesis and exosome secretion due to platinum-mediated oxidative stress were promoted. Enhanced generation of oxidative stress and activation of the ceramide pathway resulted in increased release of exosomes prompted by PtNPs. The fact that PtNPs promoted the biosynthesis and release of exosomes may contribute to our knowledge about exosomal release and biosynthesis. PtNPs could be novel agents to enhance the production of exosomes in A549 cells. The release of exosomes could have a role in the prevention and treatment of tumors. In conclusion, PtNPs may boost exosome formation *via* increased activity of the ceramide pathway and generation of oxidative stress (Gurunathan et al., 2021a).

One of the benefits of NPs in the treatment of tumors, could be the use of combined strategies to counteract MDR using formulations of anti-tumor drugs combined with NPs. PtNPs are thermo-plasmonic, thermally stable, effective and non-toxic agents. Additionally, PtNPs can be produced from naturally occurring biologic molecules in a non-toxic and environmental-friendly manner.

Doxorubicin (DOX) is a common chemotherapeutic agent used in clinical proactice, which is often investigated for combination therapy (Frederick et al., 1990; Zhao et al., 2015). One of the benefits of administering a combination therapy of chemotherapeutic drugs and NPs is that they affect more than one pathways, giving a synergistic benefit produced by two different molecules in the treatment of cancer, thus overcoming drug resistance and reducing side effects. Previous studies have shown that a combination of salinomycine (a polyether ionophore antibiotic) and AgNPs promoted cellular autophagy and apoptosis in ovarian cancer cells (Zhang and Gurunathan, 2016). It was also shown that AgNPs could enhance gemcitabine-mediated apoptosis in human ovarian cancer cells (Yuan and Gurunathan, 2017). Moreover, a recent study showed that cellular apoptosis in human breast cancer was enhanced by a combination of tubastatin-A (a histone deacetylase inhibitor) and PdNPs (palladium NPs) *via* affecting multiple cellular pathways (Yuan and Gurunathan, 2017). Another histone deacetylase inhibitor MS-375 plus AgNPs was reported to stimulate the accumulation of autophagosomes, cause mitochondrial dysfunction, activate caspase 9/3, as well as downregulate anti-apoptotic genes and upregulate pro-apoptotic genes in human basal epithelial alveoli cells (Gurunathan et al., 2018). In a study by Gurunathan et al., the anti-tumor activity of a combination of DOX and PtNPs in human bone osteosarcoma epithelial cells was assessed (Gurunathan et al., 2019a). They prepared the PtNPs using the natural product tangeretin (a pentamethoxyflavone) and demonstrated their thermal stability, non-toxicity, and thermoplasmonic properties. The combination benefits of DOX and PtNPs on multiple variables were assessed, including cell proliferation, viability and morphology, generation of ROS (reactive oxygen species), nitric oxide production, lipid peroxidation, antioxidants, protein carbonyl content, MMP (mitochondrial membrane potential), ATP levels, expression of antiapoptotic and proapoptotic genes, and genes involved in DNA damage and repair. The DOX and PtNPs combination remarkably suppressed proliferation and viability of U2OS cells in a dose-dependent manner, and increased lactate dehydrogenase release. The production of malondialdehyde and ROS, carbonylated protein and nitric oxide levels were all increased. Mitochondrial dysfunction was shown by lower levels of MMP and ATP, as well as higher expression of pro-apoptotic genes and lower expression of anti-apoptotic genes. The fact that oxidative stress plays a key role in both genotoxicity and cytotoxicity was confirmed by lower levels of various antioxidants. Moreover, DOX plus PtNPs enhanced levels of 8-oxo-G and 8-oxo-dG in addition to provoking DNA damage and expression of DNA repair genes (Gurunathan et al., 2019a).

Analysis of biodistribution of PtNPs showed that no Pt was detected in plasma 24 h after injection indicating that PtNPs had been either excreted from the system or deposited in organs or tissues. Organ accumulation data shows a high deposition of Pt in the liver and spleen after 24-h exposure and an increase after 3 weeks. This doubling of Pt in the liver would indicate that the micelles are deposited or accumulated in other organs or tissues, and then transported to the liver over the course of weeks, where they are unable to be metabolically processed for excretion or removal. The increase in total Pt in the spleen, a portion of the lymphatic system, suggests that some particles are scavenged though the lymph system and trafficked to the spleen. Tumor-bearing mice, analyzed 24 h post-injection, show lower accumulation amounts relative to non-tumored mice. The tumors accumulated approximately 3% of the injected Pt, which may account for the lower distributions in the remaining organs (Brown et al., 2018).

Table 1 shows some reports of the anti-tumor effects of PtNPs in several malignancies.

**TABLE 1:** Some therapeutic effects of PtNPs against various cancers.

Cancer	Type of platinum NPs	Particle size	Cell line or animal model	Ref
Bone	PtNPs	30 nm	U2OS	Gurunathan et al. (2019b)
Brain	PtNPs	20–110 nm	A549, MDA-MB-231, LNCaP	Gurunathan et al. (2020a)
	Silver/PlatinumNPs (AgPt)	42 ± 11 nm	HDF, A375, U87	López Ruiz et al. (2020b)
	PtNP-Based Microreactors	2 nm	SH-SY5Y	Armada-Moreira et al. (2017)
	PtNPs	1–21 nm	Neuro 2 A	Manikandan et al. (2013)
	H2PtCl6/SiO2	1.7 nm	C6, male Wistar rats	López et al. (2010)
	H2PtCl6/TiO2	3.1 nm		
	AuNPs–Pt	50 nm	S1, S2, SP56	Setua et al. (2014)
Bladder	AuPdPt NPs	30 nm	Bladder cancer	Ma et al. (2015)
	Au@Pt-nanoseeds	10–50 nm	EJ	Shin et al. (2018)
Breast	PtNPs	15 nm	4T1	Gu et al. (2019)
	PtNPs	1–6 nm	HeLa, MDA-MB-23	Aygun et al. (2020)
	Au@Pt	30 nm	SKOV3(HER2+), MDA-MB-231(HER2-)	Wawrowicz et al. (2021)
	PtNPs	45 nm	MCF-7	Baskaran et al. (2017)
	Fe3O4 magnetic NPs (MNPs), PtNPs	4 nm	SKBR-3, WM-266–4	Kim et al. (2014)
	polymeric-cisplatin NP (PIMA-cisplatin)	80–140 nm	LLC, 4T1	Paraskar et al. (2010)
			Murine Lewis Lung Carcinoma	
			Murine 4T1 Breast Cancer	
			Murine Ovarian Cancer	
	PEG–PIMA–cisplatin NPs	80–140 nm	4T1**,** *in vivo*	Paraskar et al. (2011)
			Murine 4T1 breast cancer	
	PtNPs	20.12 nm	MCF-7	Şahin et al. (2018)
	Pt@Bi2Te3−PEG	80 nm	4T1	Ma et al. (2020)
	Pt (IV) prodrug loaded in polymer NPs (PSDE-co-LDI)	156 nm	A549 (cisplatin sensitive)	Ding et al. (2020)
			*In vivo* toxicity evaluation in murine breast cancer	
Cervical				Armada-Moreira et al. (2017)
	PtNPs	30–60 nm	SiHa	Alshatwi et al. (2015)
	FePt NPs	3.11 ± 0.53 nm	Vero, HeLa	Zheng et al. (2015)
Colon	AuPt NCs	20 nm	SW480, SW62	Klebowski et al. (2020)
	PtNPs	100 nm	HT29	Pelka et al. (2009)
	PtNPs	40 nm	A549	Gurunathan et al. (2021b)
	Au-Pt NPs	99.54 nm	HCT-116	Chaturvedi et al. (2021)
	Pt-NPs		Human colon carcinoma cell line	Blank et al. (2014)
	Pt/MgO NPs (platinum-doped magnesiaNPs)	30–50 nm (TEM)	A549, HT29	Al-Fahdawi et al. (2021)
		932.3 ± 22.0 nm (DLS)		
Liver	PtNPs	86 nm	K562, HepG2	Zeng et al. (2020)
			Tumor xenograft murine model	
	PtNPs	6.30 ± 2.4 nm	HuH-7	Almarzoug et al. (2020)
	PtNPs	20–40 nm	HepG2	Medhat et al. (2017)
			*In vivo* studies	
Leukemia	PtNPs	30 nm	Human Acute Monocytic Leukemia Cells	Gurunathan et al. (2020b)
Lymphoma	Pt-NPs capped with polyacrylate	—	U937**,** HH	Yoshihisa et al. (2011)
	PtNPs	—	U937	Jawaid et al. (2016)
Lung	Polymeric delivery system (PEI-PCL-PEG micelleplexes)	160 nm	Lung cancer cell line	Feldmann et al. (2020)
	Porous platinum NPs	115.6 nm	NSCLC	Li et al. (2019a)
			*In vivo* studies	
	PtNPs	4–12 nm	A549	Ullah et al. (2017)
	PtNPs	20 nm	A549	Dobrucka et al. (2019)
	HA (BPEI-SS-Pt) delivery system	160–230 nm	Human non-small cell lung cancer cells	Jia et al. (2020)
			*In vivo* tumor model	
	FePt-Cys NPs	26.4 nm	A549, H1975, LLC	Sun et al. (2018)
			*In vivo* assay	
	Carboplatin-loaded PLGA NPs	300 nm	MA148, A549, NCI-ADR/RES, MDA-MB-231	Sadhukha and Prabha, (2014)
	Liposomal NPs containing cisplatin	120–140 nm	NCI-H596, NSCLC	Yang et al. (2014)
	PtNPs	—	A549	Yogesh et al. (2016)
			*In Vivo*	
Melanoma	PtNPs	12.2 ± 0.7 nm	B16/F10	Daneshvar et al. (2020)
Ovarian	PLGA-PEGNP co-delivery of cisplatin (CP) and wortmannin	80–200 nm	Platinum resistant ovarian cancer (PROC)	Zhang et al. (2018a)
			*In vivo* anticancer efficacy	
	PtNPs	30–70 nm	SK-OV-3	Samadi et al. (2018)
	PLGA NPs containing carboplatin (CP)	222 ± 1.1 nm	OC SKOV-3	Sánchez-Ramírez et al. (2020)
	Cisplatin-loaded biodegradable	70 ± 30 nm	SKOV3	Bortot et al. (2020)
	NPs (*Cis*-NP)		Animal Studies	
	FePt NPs	80 nm	A2780	Xu et al. (2009)
				Turiel-Fernández et al. (2021)
	NPs Metal-Organic Cages (nMOC)	98.0 ± 8.2 nm	Human ovarian cancer cell lines	Wang et al. (2019a)
Prostate	Platinum prodrug Pt (IV) in melanin-like NPs (MeNPs)	73.7 nm	PC3	Zhang et al. (2018b)
			*In Vivo*	
Oral Squamous	Cisplatin encapsulated in liposomes (LPC NPs)	35 ± 0.8 nm	Squamous cell oral carcinoma	Gusti-Ngurah-Putu et al. (2019)
			*In Vivo* Toxicity Assay	

## PtNPs as Delivery System

Treatment of cancer with non-targeted drugs encounters huge difficulty since the majority of chemotherapeutic drugs are disseminated all over the body, which results in systemic toxicity and patient intolerance, therefore the treatment is often discontinued (Neha and Momin, 2021).

**FIGURE 6:**
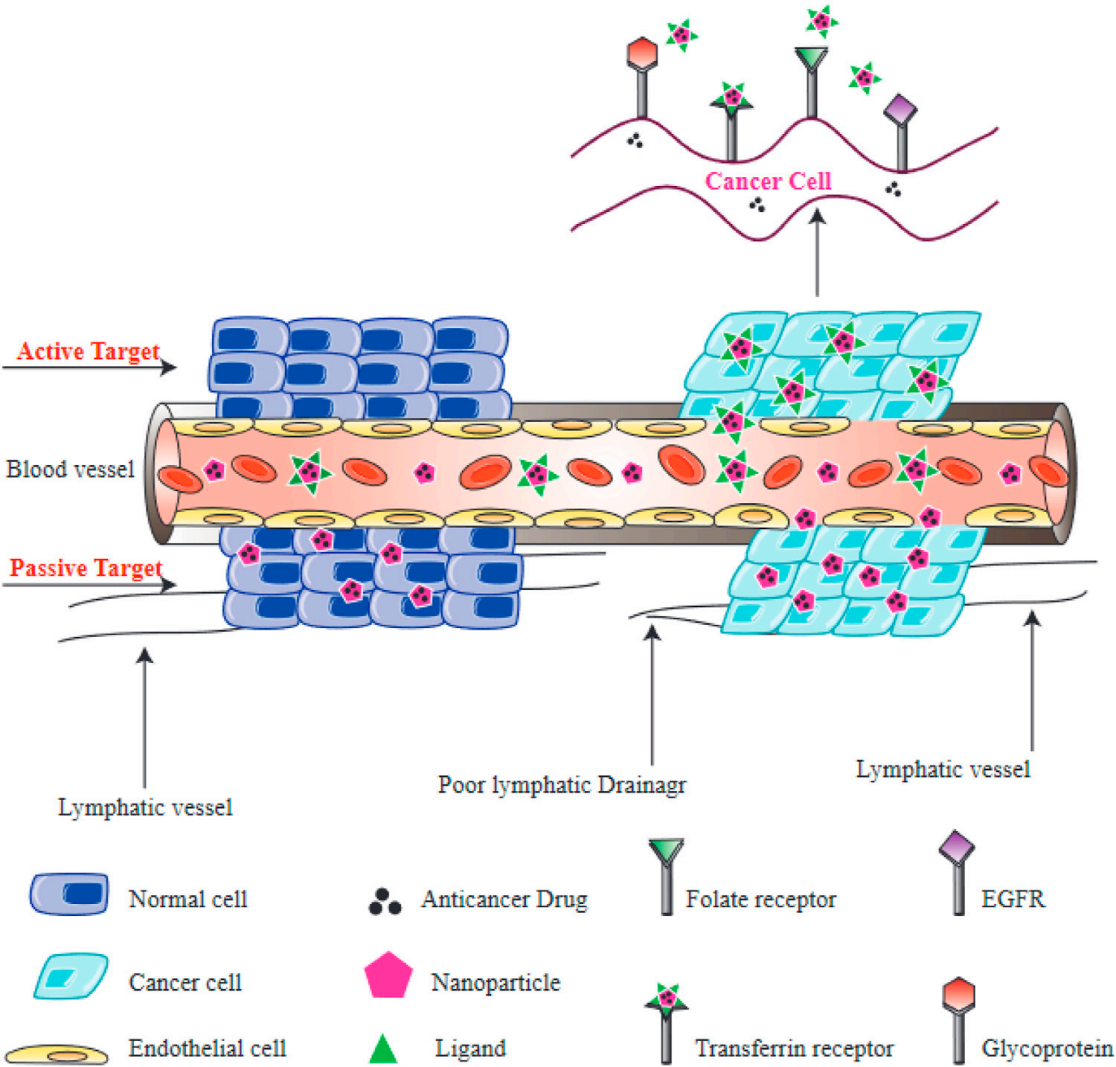
Active and passive targeting of NPs to tumors. Passive targeting of NPs is primarily mediated by the enhanced permeability and retention (EPR) effect, which depends on leaky vasculature and defective lymphatic drainage of tumors. Active targeting of NPs is mediated by interactions between receptors on cells and ligands on NPs. Folate receptors, transferrin receptors, epidermal growth factor receptor (EGFR), and glycoprotein receptors (lectins) are all found on the surface of cancer cells.

Tumor cell targeting is an important characteristic of the nanocarriers used for drug delivery, because it enhances treatment effectiveness while avoiding damage to healthy non-cancerous cells and tissues. Various studies have been performed to investigate drug delivery with targeted NPs. It is important to understand how tumors and cancer cells can interact with nano-carriers at a mechanistic level in order to rationally design efficient drug carriers. Mechanisms of targeting can be classified into two distinct categories, active and passive targeting (Figure 6).

Active targeting relies on a direct interaction occurring between a receptor and a ligand, to selectively recognize tumor cells. NPs can be equipped with certain ligands on their surface, which recognize aberrantly expressed biomolecules on the tumor cells, and therefore normal cells can be distinguished from targeted cancer cells (Shi et al., 2011; Kamaly et al., 2012). The interaction between receptors located on the surface of tumor cells and the functionalized NPs results in endocytosis, which allows the release of therapeutic agents from the internalized NPs (Farokhzad and Langer, 2009). Consequently, the process of active targeting is considered to be particularly beneficial for the delivery of macromolecular drugs, such as siRNAs or proteins. Targeting moieties which can be used to recognize these receptors, include amino acids, vitamins, peptides, monoclonal antibodies and carbohydrates (Danhier et al., 2010). Some receptors located on the surface of target cells that have been used for targeting include, glycoproteins recognized by lectins, transferrin receptor recognized by transferrin, the epidermal growth factor receptor (EGFR) recognized by epidermal growth factor, and folate receptor recognized by folic acid.

Passive targeting takes advantage of the physical properties of NPs (size, shape, and surface charge) which can interact with properties of healthy or diseased tissues. Tumor cells can rapidly proliferate, which requires the formation of new blood vessels. These form a porous network, meaning that macromolecules and NPs can leak out into the tumor, compared with healthy blood vessels in normal tissues (Carmeliet and Jain, 2000). The leaky neovasculature means that injected NPs will concentrate in tumor microenvironment after an appropriate time. Additionally, cancer tissues generally have poorly developed lymphatic drainage, resulting in the retention of NPs inside the tumor tissue and the subsequent release of their drugs into the tumor cells. Altogether, these processes lead to the enhanced permeability and retention (EPR) effect, which underlies passive targeting (Maeda, 2001). The size of the NPs may affect the EPR effect, since various studies have found that NPs with smaller sizes are more likely to leak out of tumor blood vessels compared to normal blood vessels (Torchilin, 2005; Carita et al., 2018). On the contrary, larger NPs are more likely to be removed by the immune response and the reticuloendothelial system (RES) (Sykes et al., 2014). The cancer microenvironment is also another major factor in the passive delivery of NPs. Glycolysis is considered as a typical metabolic features of tumor cells, and provides a substantial energy source for the proliferation of cancer cells (Pelicano et al., 2006). The acidic environment created by the glycolysis lowers the pH of the tumor microenvironment. Therefore, various NPs have been designed to be sensitive to pH, and can be triggered by a minimum pH level in the tumor microenvironment (Lim et al., 2018). Nevertheless, there are some drawbacks to passive targeting, including non-specific drug distribution, variations in vascular permeability in different tumors, leading to unpredictable EPR targeting (Jain, 1994).

## PtNPs as Delivery Vehicles for Anti-Cancer Agents

**FIGURE 7:**
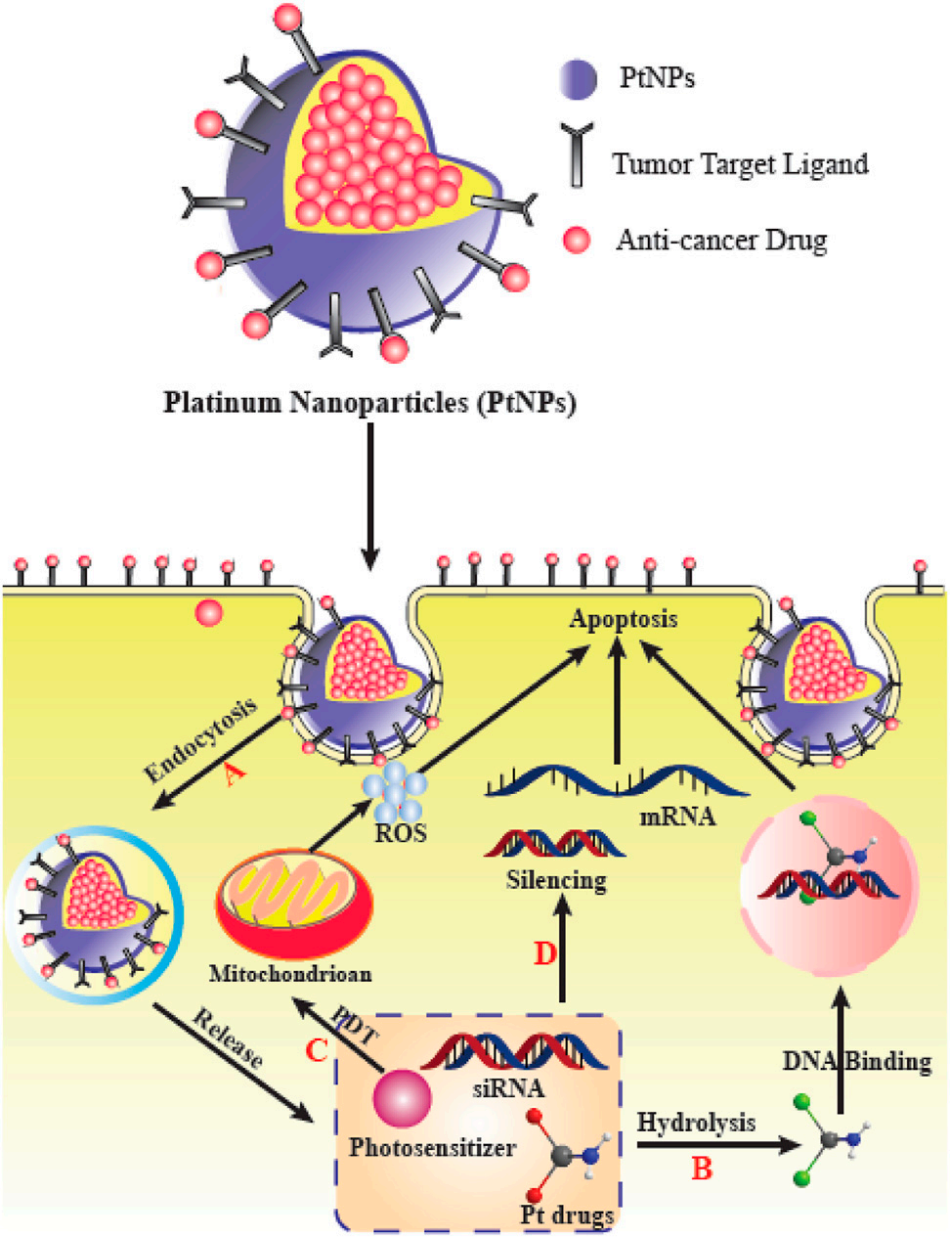
PtNPs for delivery of various therapeutic agents and their effects in cancer cells. **(A)** PtNPs improve cellular uptake *via* endocytosis to circumvent the limit of transporters. **(B)** PtNPs with other chemotherapy drugs to achieve a synergistic effect. **(C)** PtNPs with photodynamic therapy (PDT). **(D)** Co-delivery PtNPs with siRNA can silence the drug-resistance-related genes. This figure adapted from Xie et al. (2021).

Multifunctional NPs have been investigated to enhance the treatment efficiency of malignant bone tumors such as osteosarcoma (Schroeder et al., 2011; Cole et al., 2016; Jiang et al., 2017). Nanocarriers have been designed to improve the accumulation of therapeutic compounds within the vicinity of malignant bone tumors for more effective treatment (Bansal et al., 2005; Thamake et al., 2012; Heller et al., 2013; Morton et al., 2014; Zhou et al., 2016). Recent studies have shown that numerous ligands can be used to target bone tumors, such as bisphosphonates (Ramanlal Chaudhari et al., 2012; Cole et al., 2016; Yin et al., 2016), aspartic acid-related oligopeptides (Zhang et al., 2012; Wang et al., 2014), or aptamers (Liang et al., 2015a). Nevertheless, there are still issues remaining involving the safety and complex synthesis of these agents (Wang et al., 2007; Woodward et al., 2011). For example, although bisphosphonates have been commonly applied for the treatment of bone tumors and osteoporosis (Vachal et al., 2006; Body et al., 2010), they have numerous side effects such as esophageal cancer, and, atypical fractures of the femur following prolonged administration (McClung et al., 2013). As a result, developing new targeting ligands with favorable biocompatibility, simple chemistry, and more efficacy is critical for efficient treatment of malignant bone tumors. Phytic acid (PA) is a polyphosphorylated carbohydrate which is present in human diets rich in fibers, and is found in legumes and cereals (Chen, 2004; Vucenik and Shamsuddin, 2006) and is also present in the majority of mammalian species (Wilson et al., 2013). As a component of everyday foodstuffs, it has been shown to be extremely biocompatible (Chen et al., 2014). Structurally, PA contains six phosphate groups, indicating its potential bone-targeting ability similar to bisphosphonates. Moreover, PA also displays an intrinsic anti-tumor activity (Shamsuddin et al., 1997; Shamsuddin, 1999; Vucenik and Shamsuddin, 2006) (Figure 7).

Zhou et al**.** fabricated PtNPs coated with PA (PA/PtNPs) for bone-targeting, and investigated a combination of PTT (photothermal therapy) plus PA anticancer activity in malignant bone cancers (Zhou et al., 2019). As shown by *in vitro* assays, the PA/PtNPs displayed a binding affinity for hydroxyapatite and bone fragments, in addition to their intrinsic anti-tumor activity. PA was shown to remarkably promote the accumulation of PA/PtNPs in cancer-related bone lesions as shown by *in vivo* biodistribution analysis. They found that combination therapy with PA/PtNPs could effectively inhibit bone cancers and decrease osteolysis. This new and effective method to generate bone-targeted NPs with intrinsic anti-tumor activity could be used in the combined therapy of malignant bone cancers (Zhou et al., 2019).

Mesoporous PtNPs have been synthesized using surfactants as agents to control the formation of nanostructures (Teng et al., 2006; Cheong et al., 2009; Wang and Yamauchi, 2009; Wang et al., 2010; Wang et al., 2011). These NPs possess multiple binding sites for various guest molecules, thereby offering a novel alternative in cancer treatment. NP-based combination therapy with chemotherapeutic drugs plus photothermal therapy has been widely investigated. Typically, hyperthermia can be produced by laser activation of the NPs (photothermal effect) while chemotherapeutic drugs can be simultaneously delivered to the tumor in order to enhance the treatment. In one recent review, Li et al. summarized the progress in NP-based combinations of chemotherapy and photothermal therapy, including organic-based, carbon-based, and metal-based nanomaterials (Li et al., 2019b). Due to their capacity to be loaded with high amounts of drugs and good photothermal conversion, mesoporous metal nanomaterials have gained considerable interest.

Fu et al**.** prepared mesoporous PtNPs using the F127 surfactant (Fu et al., 2020). Next, PEG (polyethylene glycol) was coated on the platinum surface (PEG@Pt), and DOX was loaded by electrostatic adsorption to produce PEG@Pt/Dox. The PEG@Pt/Dox release profile was examined in PBS at pH 5.5 and 7.4. The distribution and uptake of PEG@Pt/Dox in MCF-7/ADR cells was measured by flow cytometry, TEM, and confocal imaging. The photothermal properties of PEG@Pt/Dox were investigated, and *in vitro* studies using MCF-7/ADR cells treated with PEG@Pt/Dox and irradiated with an 808 nm laser showed cytotoxicity. Dox was released predictably from PEG@Pt/Dox in PBS at pH 5.5. Confocal imaging showed that PEG@Pt/Dox was taken up by Dox-resistant breast tumor cells (MCF-7/ADR). Dox was in the cytoplasm after 1 or 12 h, and in the cell nucleus after a 24-h incubation period. Flow cytometry and TEM also confirmed cell uptake. When irradiated with an 808 nm laser, the antitumor effect was increased, and roughly 84% of tumor cells were destroyed at Dox concentrations of 8 μg/ml or more. Combination therapy was more efficient in MCF-7/ADR cells compared to the wild type MCF-7 cells. Combined therapy with photothermal and chemotherapeutic agents against drug resistant cancer cells could be provided by the multifunctional nanoplatform PEG@Pt/Dox (Fu et al., 2020).

Platinum-based anticancer drugs as well as PtNPs can both cause breakage of DNA strands inside cells (Yamagishi et al., 2013; Dasari and Tchounwou, 2014; Johnstone et al., 2014). However, different from soluble Pt-based drugs, PtNPs can also effectively scavenge hydrogen peroxide and superoxide showing an anti-oxidant activity, and can function in cancer theranostics because of their imaging capability (Kajita et al., 2007; Pelka et al., 2009; Asharani et al., 2010; Park et al., 2010). On the other hand, PtNPs have been shown to exhibit cytotoxicity, which limits their widespread use in medicine and healthcare (Gehrke et al., 2011; Yamagishi et al., 2013). Consequently, designing and testing more biocompatible PtNPs for cancer treatment remains a major issue. Moreover, the efficiency of nanomedicine for cancer may be limited by poor circulation times, and limited persistence of nano-drug conjugates and NPs within the tumor tissue. Typically, nanoconjugates are usually removed from body shortly after being recognized by human immune system. To overcome this problem, NPs are often pegylated by attachment of the Food and Drug Administration (FDA) approved PEG, thereby increasing the circulation and retention time, and reducing non-specific serum protein adsorption to avoid rapid clearance by the RES (Treuel et al., 2014; Pelaz et al., 2015).

Mukherjee et al.**,** used the borohydride reduction technique to generate PEGylated PtNPs in colloidal form at room temperature (Mukherjee et al., 2020). The PtNPs were stable in storage for longer than 2 years, and for roughly 1 week in PBS pH 7.4 and serum. The PtNPs were biocompatible in various normal cell lines *in vitro*, and a chicken egg embryo model. They then developed a PtNP-based delivery system for DOX (PtNPs-DOX). Various analytical methods were used to characterize PtNPs-DOX and the PtNPs. Numerous *in vitro* assays demonstrated that PtNPs-DOX suppressed growth of tumor cells (B16F10 and A549). Annexin-V staining confirmed that PtNPs-DOX stimulated apoptosis in tumor cells. When administered intraperitonealy (IP) in a mouse model of subcutaneous melanoma, PtNPs-DOX significantly inhibited tumor proliferation in comparison with the free drug. The anti-tumor activity was confirmed by the decreased expression of Ki-67 and SOX2 proliferation markers, and increased expression of the tumor suppressor protein p53 in malignant melanoma as shown by Western blotting and immunofluorescence. The study suggested that a drug delivery system based on PtNPs should be further investigated in anticancer nanomedicine applications (Mukherjee et al., 2020).

Numerous ligands including, peptides, antibodies, and DNA/RNA aptamers have been used to prepare targeted nanocarriers. Despite widespread use of an anti-EGFR monoclonal antibody to prepare tumor-targeted NPs, its large molecular weight and size, limits the penetration of this monoclonal antibody into the tumor microenvironment (Nida et al., 2005; Wu et al., 2006; Melancon et al., 2008; Patra et al., 2008; Yang et al., 2008; Peng et al., 2011). Meanwhile, aptamers have some advantages compared to antibodies as cancer targeting ligands, such as decreased toxicity, lack of immunogenicity, adequate tissue penetration, and better thermal stablility (Li et al., 2013). Compared to other ligands, the structure of aptamers can be easily altered to abolish undesirable interactions with proteins, to improve the blood circulation time (Farokhzad et al., 2004; McNamara et al., 2006; Javier et al., 2008; Dassie et al., 2009). Moreover, aptamers have been extensively used in the diagnosis and treatment of cancers (Ireson and Kelland, 2006; Cerchia and De Franciscis, 2010; Lee et al., 2010). The use of aptamers to transport chemotherapeutic drugs may improve the efficacy of cancer therapy. The aptamers interact with receptors located on the surface of the tumor cells in an analogous manner to antibodies. Recent research into the use of aptamers as ligands for targeted delivery of chemotherapeutic drugs has yielded promising results (Dobrovolskaia et al., 2008; Guo et al., 2013).

SPIONs (superparamagnetic iron oxide NPs) have been utilized as experimental contrast agents for MRI (magnetic resonance imaging) to diagnose tumors (Lin et al., 2008; Mailänder and Landfester, 2009; Shubayev et al., 2009; Zhang et al., 2010). Two types of SPION contrast agents, Feridex® and Resovist® have received approval by the FDA (Gandhi et al., 2006; Mailänder and Landfester, 2009). Despite the fact that SPIONs can increase contrast in MRI images, ,radioisotope-based imaging techniques including positron emission tomography usually produce stronger contrast enhancement (Tsukada, 2011). Noninvasive diagnosis of tumors requires greater signal enhancement, which can be provided by more powerful MRI contrast agents. One example is SIPPs (superparamagnetic iron platinum NPs), which were previously shown to be more effective MRI contrast agents compared to SPIONs (Taylor et al., 2011; Taylor and Webster, 2011). One study investigated the use SIPPs as MRI magnetic NP-based contrast agents. They showed that SIPP particles could be encapsulated into micelles formed from PEGylated phospholipids. These particles could selectively target prostate tumor cell lines using an anti-PSMA (prostate-specific membrane antigen) antibody *in vitro* (Taylor et al., 2011).

In a study by Taylor et al., multifunctional SIPP-PTX micelles (SPMs) were produced from biotin, fluorescein, and PEG functionalized phospholipids. They were conjugated to an antibody against PSMA and loaded with paclitaxel. These multifunctional SIPP-PTX micelles were tested for the treatment of prostate tumors in a mouse xenograft model, with the advantages of MRI and selective targeting (Taylor and Sillerud, 2012). The average diameter of the SPMs was 45.4 ± 24.9 nm and they contained 247.0 ± 33.4 μg/ml platinum, 702.6 ± 206 μg/ml paclitaxel, and 160.7 ± 22.9 μg/ml iron. Measurement of drug release, showed that approximately 50% of paclitaxel was released in serum after 30.2 h, while in saline it took twice as long. SPMs conjugated to anti-PSMA could selectively bind to C4-2 human prostate tumor cells in a binding assay, and release paclitaxel into the tumor cells *in vitro*. Free paclitaxel was shown to be more cytotoxic compared to SPMs in C4-2 cells, after 48 and 24 h incubation periods (1.6 times and 2.2 times, respectively). However, after 72 h, SPMs and paclitaxel displayed similar cytotoxicity. The MRI transverse relaxivity of SPMs was 389 ± 15.5 Hz/mM iron. SIPP micelles could generate contrast enhancement in MRI regardless of the presence of drug *in vivo*. In nude mice, the formation and proliferation of C4-2 prostate tumor xenografts was specifically inhibited by paclitaxel loaded PSMA-targeted SPMs. Besides, nude mice injected with PSMA-targeted SPMs, showed higher concentrations of both platinum and paclitaxel in the cancer tissue in comparison with nude mice injected with non-targeted paclitaxel and SPMs (Taylor and Sillerud, 2012).

Table 2 summarizes some reports of platinum NPs which have been used to deliver different drugs to different tumors.

**TABLE 2:** PtNPs used to deliver drugs or therapeutic agents to different tumors.

Cancer	Anti-cancer agent	Particle size	Cell line or animal model	Ref
Bone	Phytic acid capped platinum NPs(PA/PtNPs)	1.7 ± 1.2 nm	NIH3T3, PC-9, *In vivo* therapy of bone tumors	Zhou et al. (2019)
Brain	FePt NPs coated with oleic acid/oleylamine (OA/OA) and cysteine (Cys)	3–8 nm	U25, U87, H4	Sun et al. (2012)
Brain	l-cysteine coated FePt (FePt-Cys) NP	DLS, 245 nm	C6, SGH44, U251	Liang et al. (2015b)
		TEM, 4.8 ± 0.6 nm		
Breast	Doxorubicin-loaded PEG@Pt (PEG@Pt/Dox)	120 ± 5 nm	MCF-7/ADR	Fu et al. (2020)
Breast	Four Pt (II) complexes conjugated to iron oxide NPs	27–100 nm	MDA-MB-231**,** IGROV-1	Quarta et al. (2019)
	1. PEG-Glu-Pt-EDA			
	2. PEG-Glu-Pt-DACH			
	3. PEG-Mal-Pt-EDA			
	4. PEG-Mal- Pt-DACH			
Breast	PIMA–GA–DACH–PtNPs (polyisobutylene maleic acid copolymer with glucosaminechelates diaminocyclohexane (DACH) platinum (II)	80–250 nm	4T1, MDA-MB-231**,** CP20	Paraskar et al. (2012)
			*In vivo* 4T1 breast cancer tumor model	
Breast	Dox loadedFu-PtNPs (fucoidan-coated Pt NPs)	33 ± 3.4 nm	MCF-7/ADR	Kang et al. (2019)
			*In Vivo*	
Breast	GO-NP-Pt (graphene oxide functionalized with platinum NPs)	2–19 nm	MCF-7**,** LNCaP**,** Hela B**,** HepG2**,** SW480**,** HT29**,** HCT116**,** Colo205	Kutwin et al. (2019)
Cervical	Apt-Pt NPs (EGFR-targeted albumin-cisplatin NPs with EGFR aptamer)	80 nm	Hela	Chen et al. (2016)
			*In Vivo* Cervical Cancer Model	
Cervical	PtAu NRs (PtAu NRs nanoraspberries)	10 nm	SW480, SW620	Depciuch et al. (2019)
Colon	cisPt@BRNP (cisplatin-chelated BR-based NP)	192 ± 88 nm	HT-29	Lee et al. (2017)
			*In Vivo*	
Colon	polyphenols capped Pt NPs	10–70 nm	HCT 116	Yang et al. (2017)
Colon	NANO-Pt-Pan (DACH-Pt (II)-modified panitumumab)	120–155 nm	HT29, Caco2, HCT116	Tsai et al. (2017)
			*In Vivo* Antitumor Efficacy	
Liver	PtNPs (Peptide-stabilized platinum NPs)	2.5 nm	HepG2	Shoshan et al. (2019)
Liver	GA-ALG@Pt NPs (Pt (IV) prodrug-loaded glycyrrhetinic acid (GA)-modified alginate NP)	141.9 nm	HepG2	Wang et al. (2019b)
Lung	GP-NA (GEM-grafted copolymers (PEG-b-P (LL-g-GEM)), pH-sensitive polypeptides (OAPI), and USPtNs)	165.4 ± 2.6 nm	A549, NCI-H1299	Shi et al. (2020)
			*In vivo* anti-cancer efficacy	
Lung	PEG-PtCNPs (platinum-doped, carbon NPs)	18.7 ± 4.6 nm	A549 (or U14)	Bao et al. (2018)
			*In vivo* toxicity assay	
Lung	Platinum Encapsulated Chitosan NPs	230–270 nm	A549, A549l	Nascimento et al. (2015)
Lung	HA-GEM/CH-Pt NPs	200 nm	NCl-H460	Zhang et al. (2017)
			*In vivo* antitumor efficiency	
Lung	CDDP-NPs (Cisplatin-loaded PLGA)	36.7 ± 8.1 nm	LLC, HeLa	Shi et al. (2015)
			Animal testing	
Lung	NP-TPGS-Pt	85.3 nm	Pt-resistant A549/DDP	Tsai et al. (2018)
Melanoma	PtNPs-DOX	40–45 nm	NIH-3T3, A549**,** B16F10	Mukherjee et al. (2020)
			*In vivo* tumor model	
Ovarian	PPNPsiRNA (photoactivatedpolyprodrug NP system)	110 nm	A2780	Zhang et al. (2020)
Prostate	Pt-NP-Apt polymer NPs	140 nm	PSMA-overexpressing LNCaP	Dhar et al. (2008)
			PSMA^**-**^ PC3	
Prostate	Pt-PLGA-b-PEG-Apt-NP	150 ± 15 nm	LNCaP	Dhar et al. (2011)
			*In vivo* anticancer efficacy	
Oral Squamous	PtNCP (platinum nanocomposite beads)		Oral squamous cell carcinoma cell lines	Tanaka et al. (2019)
			Animals tumor xenograft model	

## Conclusion

Platinum-based biomolecules have attracted much interest for several years. For example, platinum-derived therapeutic drugs have been extensively investigated to destroy tumor cells. However, these chemotherapeutic agents also have several problems, as they cause significant dose-dependent acute and chronic side effects due to their non-selective action on both tumor and healthy cells.

Recently, the development of nanomaterials with eco-friendly properties has played a major role in many different fields, such as energy applications, electronics, biomedical engineering, and drug delivery. Platinum NPs are one type of metal NPs, which have many advantages, such as anti-tumor and anti-bacterial activity, making them appropriate candidates for a wide range of pharmaceutical applications. Because PtNPs display remarkable behavior in a variety of applications, methods for their synthesis and characterization have become increasingly important. Numerous synthetic routes for PtNPs have been described, such as microemulsion, colloidal systems, sol–gel, reduction, sonochemical methods, electrochemical synthesis, and methods based on microbial and plant extracts. Because of their shape and size-dependent catalytic and optical properties, functional PtNPs have attracted considerable attention in recent years as theranostic and therapeutic platforms. These features have the potential to circumvent some drawbacks of conventional pharmaceuticals, such as unfavorable side effects, imperfect selectivity, and undesirable pharmacokinetics. Some current efforts were done to synthesize more biocompatible platinum nanoparticles which are hemocompatible (Mukherjee et al., 2021).

The vast amount of basic research on platinum coordination complexes has produced. Over the past years, several thousand new molecules for preclinical screening and 28 compounds which have entered clinical development. The goals of these research activities have been to identify compounds with superior efficacy, reduced toxicity, lack of cross-resistance or improved pharmacological characteristics as compared with the parent compound, cisplatin. Whereas carboplatin is an analogue with an improved therapeutic index over that of cisplatin, new compounds clearly more active than or non-cross-resistant with cisplatin have not yet been identified (Lebwohl and Canetta, 19901998). Although, there have been a few platinum-based nanomaterials, which have been suggested to be useful in cancer therapy. In addition, functional PtNPs have been shown to trigger apoptosis in cancer cells *via* a range of target-specific pathways. The development of new enzyme-free sensors for platinum-based nanomaterials with good selectivity, high sensitivity, portability, *in vivo* detection, and commercial application, is therefore the key direction of future research and development.
